# Accuracy and Survival Outcomes after National Implementation of Sentinel Lymph Node Biopsy in Early Stage Endometrial Cancer

**DOI:** 10.1245/s10434-023-14065-3

**Published:** 2023-08-26

**Authors:** Silvia Cabrera, Natalia R. Gómez-Hidalgo, Virginia García-Pineda, Vicente Bebia, Sergi Fernández-González, Paula Alonso, Tomás Rodríguez-Gómez, Pere Fusté, Myriam Gracia-Segovia, Cristina Lorenzo, Enrique Chacon, Fernando Roldan Rivas, Octavio Arencibia, Marina Martí Edo, Soledad Fidalgo, Josep Sanchis, Pablo Padilla-Iserte, Manuel Pantoja-Garrido, Sergio Martínez, Ricard Peiró, Cecilia Escayola, M. Reyes Oliver-Pérez, Cristina Aghababyan, Carmen Tauste, Sara Morales, Anna Torrent, Jesus Utrilla-Layna, Francesc Fargas, Ana Calvo, Laura Aller de Pace, Antonio Gil-Moreno, on behalf of the Spain-GOG and the MULTISENT Study Group

**Affiliations:** 1 Gynecologic Oncology Unit, Hospital Vall d’Hebron, Vall d’Hebron Barcelona Hospital Campus, Universitat Autònoma de Barcelona (UAB), Barcelona, Spain; 2 Gynecologic Oncology Unit, La Paz University Hospital, Madrid, Spain; 3 Gynecologic Oncology Unit, Hospital Universitari de Bellvitge, Barcelona, Spain; 4 Department of Obstetrics and Gynecology, Hospital Universitario Gregorio Marañón, Madrid, Spain; 5 Department of Obstetrics and Gynecology, Hospital Virgen de la Victoria, Málaga, Spain; 6 Gynecologic Oncology Unit, Hospital Clínic i Provincial de Barcelona, Barcelona, Spain; 7 Department of Obstetrics and Gynecology, Hospital Universitario Nuestra Señora de Candelaria, Tenerife, Spain; 8 Department of Obstetrics and Gynecology, Clinica Universidad de Navarra, Pamplona, Spain; 9 Department of Obstetrics and Gynecology, Hospital Clínico Universitario de Zaragoza, Zaragoza, Spain; 10 Department of Obstetrics and Gynecology, Hospital Universitario Gran Canarias Dr. Negrín, Las Palmas, Spain; 11 Department of Obstetrics and Gynecology, Hospital General Universitario de Ciudad Real, Ciudad Real, Spain; 12 Department of Obstetrics and Gynecology, Hospital Universitario Central de Asturias, Oviedo, Spain; 13 Gynecologic Oncology Unit, Instituto Valenciano de Oncologia (IVO), Valencia, Spain; 14 Gynecologic Oncology Unit, Hospital Politécnico Universitario La Fe, Valencia, Spain; 15 Department of Obstetrics and Gynecology, Hospital Universitario Virgen Macarena, Sevilla, Spain; 16 Department of Gynecology, Hospital Universitari Germans Trias i Pujol, Badalona, Spain; 17 Department of Obstetrics and Gynecology, Hospital General de Catalunya, Barcelona, Spain; 18 Department of Obstetrics and Gynecology, El Pilar Quiron, Barcelona, Spain; 19 Gynecologic Oncology Unit, Department of Obstetrics and Gynecology, Hospital Universitario 12 de Octubre, 12 de Octubre Research Institute (i+12), Complutense University of Madrid, Madrid, Spain; 20 Department of Obstetrics and Gynecology, Hospital General Universitario de Valencia, Valencia, Spain; 21 Department of Obstetrics and Gynecology, Hospital Universitario de Navarra, Pamplona, Spain; 22 Department of Obstetrics and Gynecology, Hospital Infanta Leonor, Madrid, Spain; 23 Department of Obstetrics and Gynecology, Hospital Universitari Son Espases, Palma de Mallorca, Spain; 24 Department of Obstetrics and Gynecology, Fundación Jimenez Diaz, Madrid, Spain; 25 Department of Obstetrics and Gynecology, Hospital Universitari Quirón Dexeus, Barcelona, Spain; 26 Department of Obstetrics and Gynecology, Hospital Universitari Arnau de Vilanova, Lleida, Spain; 27 Department of Obstetrics and Gynecology, Hospital Marqués de Valdecilla, Santander, Spain

## Abstract

**Background:**

Sentinel lymph node (SLN) biopsy has recently been accepted to evaluate nodal status in endometrial cancer at early stage, which is key to tailoring adjuvant treatments. Our aim was to evaluate the national implementation of SLN biopsy in terms of accuracy to detect nodal disease in a clinical setting and oncologic outcomes according to the volume of nodal disease.

**Patients and Methods:**

A total of 29 Spanish centers participated in this retrospective, multicenter registry including patients with endometrial adenocarcinoma at preoperative early stage who had undergone SLN biopsy between 2015 and 2021. Each center collected data regarding demographic, clinical, histologic, therapeutic, and survival characteristics.

**Results:**

A total of 892 patients were enrolled. After the surgery, 12.9% were suprastaged to FIGO 2009 stages III–IV and 108 patients (12.1%) had nodal involvement: 54.6% macrometastasis, 22.2% micrometastases, and 23.1% isolated tumor cells (ITC). Sensitivity of SLN biopsy was 93.7% and false negative rate was 6.2%. After a median follow up of 1.81 years, overall surivial and disease-free survival were significantly lower in patients who had macrometastases when compared with patients with negative nodes, micrometastases or ITC.

**Conclusions:**

In our nationwide cohort we obtained high sensitivity of SLN biopsy to detect nodal disease. The oncologic outcomes of patients with negative nodes and low-volume disease were similar after tailoring adjuvant treatments. In total, 22% of patients with macrometastasis and 50% of patients with micrometastasis were at low risk of nodal metastasis according to their preoperative risk factors, revealing the importance of SLN biopsy in the surgical management of patients with early stage EC.

Endometrial cancer (EC) is the most diagnosed gynecological malignancy in developed countries,^1^ and surgery remains the cornerstone of its treatment. Surgical treatment is especially important in early stage disease, when it also allows for a proper stratification of risk of recurrence and helps to tailor adjuvant therapies.^2^

Systematic lymphadenectomy in early stage EC has been widely questioned due to the lack of survival impact of this strategy reported in different prospective trials.^3,4^ Nevertheless, the need to know lymph node status as the most important prognostic factor in these patients brought the adoption of sentinel lymph node (SLN) biopsy as a new technique to assess lymph node disease in patients with early stage EC.^2,5^ Several prospective trials have reported an excellent accuracy of SLN biopsy in detecting lymph node disease,^6–10^ describing the technical issues implied in achieving a higher overall and bilateral detection and bringing to the fore the importance of performing ultrastaging of the specimens to increase the detection of disease and equalize, or even increase, the sensitivity of systematic lymphadenectomy.

Ultrastaging consists of the ultra-sectioning of SLN and its immunohistochemistry staining to improve the detection of low-volume disease. Several protocols for ultrastaging have been reported,^11,12^ and recently one-step nucleic acid amplification (OSNA) has emerged as a new strategy to detect node disease with high accuracy.^13,14^ Low-volume disease, a term that includes micrometastases and isolated tumor cells (ITC), represents more than 50% of the nodal disease diagnosed in endometrial cancer when using SLN biopsy.^7,8,15^ The clinical significance of these small amounts of malignant cells in SLN has been assessed,^15–19^ as it supposes a new phenomenon that needs to be elucidated at the time of clinical implementation of SLN biopsy in EC management. Previous reports suggest that isolated tumor cells (ITC, clusters of malignant cells less than 0.2 mm^20^) are usually present in patients with well-differentiated endometrioid histology and limited myometrial invasion,^21^ entailing no impact on recurrence-free or overall survival of patients with EC. Nevertheless, micrometastases (node metastases that range between 0.2 and 2 mm^20^) show worse prognosis than node-negative patients in several studies.^16,22^

We present the results of a retrospective, consecutive, multicenter, national cohort of early-stage EC patients, who received SLN biopsy as a part of their surgical protocol. Our primary objective was to evaluate the accuracy of SLN biopsy technique to detect nodal disease in a clinical setting, assessing factors affecting the detection of disease in the SLN. Secodary objective was to analyse the oncologic outcomes of patients according to the volume of nodal disease.

## Patients and Methods

### Cohort Selection

A total of 29 Spanish centers participated in the setting-up of a retrospective, consecutive, multicenter, national registry including patients older than 18 years of age diagnosed with endometrial cancer (of any grade or histology) or endometrial atypical hyperplasia, operated on between January 2015 and April 2021 with preoperative clinical stage I–II EC FIGO 2009 and who had undergone SLN biopsy as a part of their surgical protocol (MULTISENT registry). From this national database, we selected patients with final biopsy of adenocarcinoma with at least one SLN detected during surgery. Patients with suspected clinical or radiological lymph node disease, advanced stage at diagnosis, those who received neoadjuvant therapy prior to surgery, or histology of uterine sarcomas were excluded. Every participating center obtained their institutional review board (IRB) approval.

### Data Extraction

Each participating center collected retrospective data regarding demographic, clinical, histologic, therapeutic, and survival characteristics. SLN biopsy was performed using different tracers [indocyanine green (ICG) alone or combined with 99m-Technetium (99mTC), or 99mTC alone or combined with blue dyes] and different sites of injections (cervical, uterine, and both) according to each center protocol. Intraoperative frozen section of SLN or uterus were performed at surgeon’s discretion. SLN found negative in Hematoxilin/Eosin (H/E) routine study were processed according to each center’s ultrastaging protocol, although minimum ultrastaging protocol should include ultrasectioning and inmunohystochemistry (IHC) staining of SLN or OSNA analysis. Disease was classified according to its volume into macrometastases (tumor ≥ 2.0 mm in diameter), micrometastases (tumor cell aggregates between 0.2 and 2.0 mm in diameter), or isolated tumor cells (ITC, individual tumor cells or aggregates < 0.2 mm in diameter and < 200 cells).^20^ Some centers performed OSNA analysis of the specimen, and the volume of the disease was reported as follows: absence of copies of CK19 mRNA were considered negative nodes; values < 250 copies/µL of CK19 mRNA were considered ITC; values between 250 and 4999 copies/µL were considered micrometastases; and values ≥ 5000 copies/µL were considered macrometastases.^23^ For calculating accuracy rates of SLN biopsy we used as reference standard the pathologic result of pelvis ± aortic staging lymphadenectomy performed on the same patient. SLNs were considered positive when any size of disease was diagnosed through H/E or ultrastaging, although patients with ITCs only on SLN [pN0(i+)] were classified as node negative for FIGO 2009 staging and patients with micrometastases [pN1(mic)] or macrometastases (pN1) were considered node positive.

All variables were recorded in a Research Electronic Data Capture (REDCap)^24^ electronic database, designed for this study and hosted at Vall d’Hebron Institute of Research (VHIR). After the inclusion of the patient, the investigators assigned a preoperative risk of lymph node disease according to preoperative imaging and diagnostic biopsy. The allocation was as follows: low risk (preoperative stage IA and endometrioid low grade), high risk (preoperative stage IB or II and endometrioid high grade, or any non-endometrioid histology), and intermediate risk (preoperative stage IB or II and endometrioid low grade, or preoperative stage IA and endometrioid high grade).

### Statistical Analysis

Stata statistical program (version 14.2) was used for data analysis. Continuous variables were expressed as median and interquartile range (IQR) and were compared using the Kruskal–Wallis test. Categorical variables were expressed as frequencies and percentages and compared using the *χ*^2^ test or the Fisher’s exact test. All tests were two-tailed. Imputation of missing values was not performed. Oncologic outcomes were analyzed using the Kaplan–Meier method and the log-rank test. Univariate and multivariate adjusted logistic regression models were used for the comparison between groups. For the construction of the multivariate model, a selection method based on maximum likelihood estimation and Akaike information criterion (AIC) was used, considering all relevant variables related to the primary end point.

## Results

### Patient and Tumor Characteristics

**Fig. 1:**
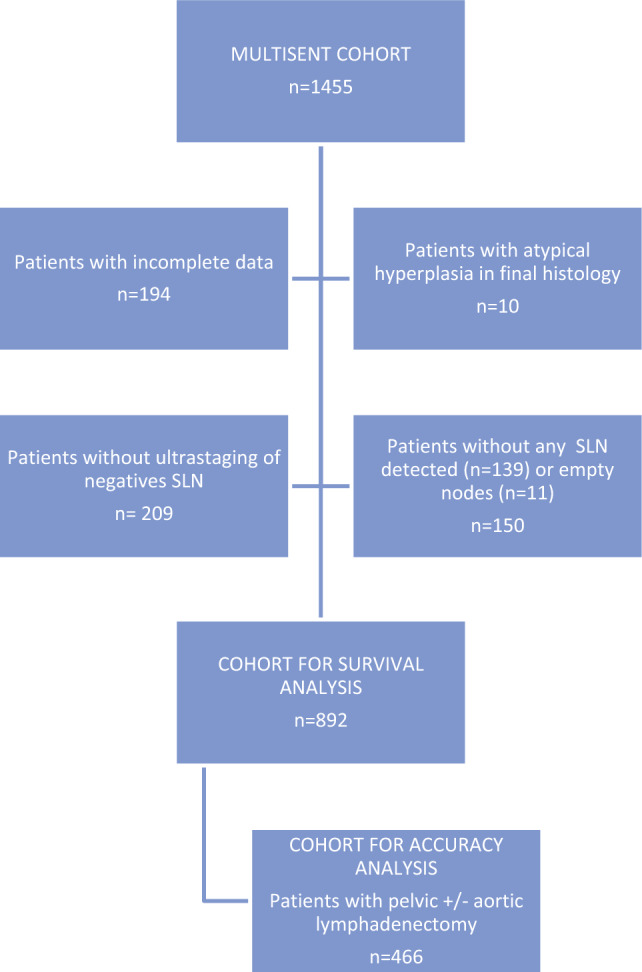
Flow diagram for cohort selection. SLN: sentinel lymph node

Out of the 1455 patients included in MULTISENT registry, 194 patients had insufficient data to be considered for this study, 10 patients presented final histology of endometrial atypical hyperplasia, therefore not meeting inclusion criteria, and 150 additional patients were not included because no SLN was detected after the performance of the technique. Patients without ultrastaging performed on negative SLN were also excluded (209 patients). Finally, 892 patients were considered for survival analysis. Out of them, 466 patients had at least unilateral pelvic lymphadenectomy and were included for accuracy analysis (Fig. 1).

Median age at diagnosis was 62.5 (IQR 55.7–70.4) years, and mean body mass index was 28.9 (IQR 25.4–33.6) kg/m^2^. A total of 87% of patients had endometrioid histology at final pathology report, and 78.6% were low grade (G1–2). Almost half of the cohort (48.8%) was considered at preoperative low risk of lymph node metastases, and they were exclusively staged with SLN biopsy. In addition, 47.4% of the patients received a bilateral lymphadenectomy in association to SLN, and 25.5% underwent an aortic lymphadenectomy as well. Regarding SLN technique, 73.2% of patients were mapped using ICG alone or in combination with 99mTc, and 95.8% received a cervical injection.

In total, 115 patients (12.9%) were suprastaged to FIGO 2009 stages III or IV after surgery, 65 patients (7.3%) were staged as FIGO 2009 IIIC1, and 17 patients (1.9%) as FIGO 2009 IIIC2 after surgery, while 108 patients (12.1%) had nodal disease; 59 with macrometastasis (54.6%) and 49 (45.3%) with low-volume disease only. Among the latter, 24 had micrometastases and 25 ITCs. Excluding patients with ITCs, 9.3% of patients included had positive SLNs. Patients with macrometastases were older and showed a significant higher proportion of high-grade tumors, bigger tumoral size, and more lymphovascular space invasion (LVSI) (Table 1).

**Table 1:** Clinicopathological and surgical characteristics of patients

Variable [mean (SD), median (IQR), or *n* (%)]	pN0 (*n* = 784)	pN0(i+) (*n* = 25)	pN1(mi) (*n* = 24)	pN1 (*n* = 59)	Total (*n* = 892)	*p* value
Age (years)	62.1 (55.4–70.1)	63.7 (57.4–65.9)	61.8 (56.5–72.4)	66.7 (61.4–72.6)	62.5(55.7–70.4)	0.015
BMI (kg/m^2^)	29.2 (25.4–33.4)	28.8 (26.1–32.5)	29.3 (24.3–35.8)	28.7 (24.8–34.2)	28.9 (25.4–33.6)	0.931
Preoperative risk groups for LN involvement						
Low risk	401 (51.2)	9 (36.0)	12 (50.0)	13 (22.0)	435 (48.8)	< 0.001
Medium risk	258 (32.9)	14 (56.0)	8 (33.3)	23 (39.0)	303 (34.0)	
High risk	101 (12.9)	2 (8.0)	3 (12.5)	23 (39.0)	129 (14.5)	
Not assigned	24 (3.1)	0 (0.0)	1 (4.2)	0 (0.0)	25 (2.8)	
Pelvic lymphadenectomy						
No	397 (50.6)	12 (48.0)	10 (41.7)	8 (13.6)	427 (47.9)	< 0.001
Bilateral	349 (44.5)	13 (52.0)	13 (54.2)	48 (81.4)	423 (47.4)	
Unilateral	38 (4.9)	0 (0.0)	1 (4.2)	3 (5.1)	42 (4.7)	
Aortic lymphadenectomy	184 (23.5)	6 (24.0)	6 (25.0)	31 (52.5)	227 (25.5)	< 0.001
Histology						
Endometrioid	691 (88.1)	22 (88.0)	21 (87.5)	42 (71.2)	776 (87.0)	0.057
Serous	50 (6.4)	2 (8.0)	3 (12.5)	13 (22.0)	68 (7.6)	
Clear cell	9 (1.2)	0 (0.0)	0 (0.0)	0 (0.0)	9 (1.0)	
Mixed	11 (1.4)	1 (4.0)	0 (0.0)	1 (1.7)	13 (1.5)	
Mucinous	4 (0.5)	0 (0.0)	0 (0.0)	0 (0.0)	4 (0.5)	
Undifferentiated	5 (0.6)	0 (0.0)	0 (0.0)	0 (0.0)	5 (0.6)	
Carcinosarcoma	14 (1.8)	0 (0.0)	0 (0.0)	3 (5.1)	17 (1.9)	
Grade						
G1	324 (41.3)	11 (44.0)	8 (33.3)	15 (25.4)	358 (40.1)	0.013
G2	301 (38.4)	10 (40.0)	12 (50.0)	20 (33.9)	343 (38.5)	
G3	149 (19.0)	4 (16.0)	4 (16.7)	24 (40.7)	181 (20.3)	
Unknown	10 (1.3)	0 (0.0)	0 (0.0)	0 (0.0)	10 (1.1)	
LVSI						
No	653 (83.3)	17 (68.0)	11 (45.8)	13 (22.0)	694 (77.8)	< 0.001
Yes	115 (14.7)	8 (32.0)	12 (50.0)	44 (74.6)	179 (20.1)	
Unknown	16 (2.0)	0 (0.0)	1 (4.2)	2 (3.4)	19 (2.1)	
Max tumor diameter (mm)	25 (15–35)	22.5 (6–35)	30 (10–37)	31.5 (20–50)	25 (15–35)	0.024
Lymphadenectomy, nodes per patient						
Pelvic (median, IQR)	11 (7.5–15)	10 (9–12)	12.5 (7–16)	12 (9–16)	11 (8–15)	0.501
Aortic (median, IQR)	10 (5–15)	5.5 (4–9)	8 (5–16)	11 (6–14)	9.5 (5–15)	0.302
FIGO stage (2009)						
IA	501 (63.9)	13 (52.0)	0 (0.0)	0 (0.0)	514 (57.6)	< 0.001
IB	213 (27.2)	8 (32.0)	0 (0.0)	0 (0.0)	221 (24.8)	
II	41 (5.2)	1 (4.0)	0 (0.0)	0 (0.0)	42 (4.7)	
IIIA	15 (1.9)	3 (12.0)	0 (0.0)	0 (0.0)	18 (2.0)	
IIIB	10 (1.3)	0 (0.0)	0 (0.0)	0 (0.0)	10 (1.1)	
IIIC1	0 (0.0)	0 (0.0)	24 (100.0)	41 (69.5)	65 (7.3)	
IIIC2	0 (0.0)	0 (0.0)	0 (0.0)	17 (28.8)	17 (1.9)	
IVB	4 (0.5)	0 (0.0)	0 (0.0)	1 (1.7)	5 (0.6)	
Tracer used for SLN detection						
ICG	348 (44.4)	11 (44.0)	9 (37.5)	23 (39.0)	391 (43.8)	0.936
ICG + 99mTc	229 (29.2)	6 (24.0)	9 (37.5)	18 (30.5)	262 (29.4)	
99mTc ± blue dye	205 (26.2)	7 (28.0)	6 (25.0)	18 (30.5)	236 (26.5)	
Unknown	2 (0.3)	1 (4.0)	0 (0.0)	0 (0.0)	3 (0.3)	
Tracer injection method						
Cervix	733 (93.5)	23 (92.0)	24 (100.0)	53 (89.8)	833 (93.4)	0.109
Cervix + uterus	15 (1.9)	1 (4.0)	0 (0.0)	5 (8.5)	21 (2.4)	
Uterus	30 (3.8)	0 (0.0)	0 (0.0)	1 (1.7)	31 (3.5)	
Unknown	6 (0.8)	1 (4.0)	0 (0.0)	0 (0.0)	7 (0.8)	

*SLN* sentinel lymph node, *LVSI* lymphovascular space invasion, *ICG* indocyanine green, *99mTc* 99m-Technetium, *pN0* pathologic node negative patients, *pN0*(*i+*) pathologic isolated tumor cell in SLN, *pN1*(*mi*) pathologic micrometastasis in SLN, *pN1* pathologic macrometastasis

### SLN Accuracy for Detecting Nodal Disease

The overall sensitivity of SLN technique was 92.5% and the overall false negative rate was 7.5%. There were six patients reported as false negative cases of SLN technique in the overall results. Two of them presented negative bilateral pelvic SLN but had isolated aortic metastases. One patient presented positive pelvic lymph nodes in a non-mapped hemipelvis and was correctly diagnosed after performing the selective pelvic lymphadenectomy (MSKCC algorithm^5^). The three remaining cases correspond to true false negative cases of the technique, as they had negative pelvic SLN but presented nodal disease in the same hemipelvis. The algorithm sensitivity was 93.7% and the algorithm false-negative rate dropped to 6.2%, including patients with isolated aortic metastases. Two patients had isolated aortic metastases, corresponding to one patient with endometrioid grade 2 tumor with bilateral and negative pelvic SLN and another patient with serous histology, who received bilateral pelvic SLN biopsy and pelvic lymphadenectomy, both negative. Thus, the rate of isolated aortic metastases in our cohort was 1.6%. It was higher in patients with high-grade endometrioid or non-endometrioid histology than in patients with low-grade endometrioid histology (2.8% vs. 0.7%, *p* = 0.35) (Table 2).

**Table 2:** Accuracy of SLN biopsy for detecting nodal disease

Overall accuracy of SLN biopsy					
SLN status per patient, *n* (%)	pN0 (*n* = 784)	pN0(i+) (*n* = 25)	pN1(mi) (*n* = 24)	pN1 (*n* = 59)	Total (*n* = 892)
Positive	0	25 (100)	24 (100)	53 (89.3)	102 (11.4)
ITC	0	25 (100)	0	1 (1.7)	26 (2.9)
Micrometastasis	0	0	24 (100)	6 (10.2)	30 (3.4)
Macrometastasis	0	0	0	46 (77.9)	46 (5.2)

SLN accuracy, *n* (%) (over the patients who received pelvic ± aortic LND)	pN0 (*n* = 386)	pN0(i+) (*n* = 13)	pN1(mi) (*n* = 14)	pN1 (*n* = 53)	Total (*n* = 466)
True positive	0	13 (100)	14 (100)	47 (88.7)	74 (15.9)
False negative	0	0	0	6 (11.3)	6 (1.3)
True negative	386 (100)	0	0	0 (0.0)	386 (82.8)
Overall sensitivity: 92.5 % (74/80); overall FNR: 7.5% (6/80)					

Assessment of false negative cases											
Case	Pelvic mapping	Number of SLN	Tracer	Injection site	Pelvic LND	Aortic LND	Pelvic positive nodes	Aortic positive nodes	Total nodes	Histology	Classification
1	Bilateral pelvic	2	ICG	Cervix	Bilateral	Yes	6	1	14	Serous	False negative
2	Bilateral pelvic	3	ICG	Cervix	Bilateral	Yes	1	1	38	Serous	False negative
3	Unilateral pelvic (right)	1	ICG	Cervix	Bilateral	No	2 (right)	–	5	Endometrioid G3	False negative
4	Bilateral pelvic	2	ICG	Cervix	No	Yes	0	1	5	Endometrioid G2	Isolated aortic metastasis
5	Bilateral pelvic	3	ICG	Cervix	Bilateral	Yes	0	1	21	Serous	Isolated aortic metastasis
6	Unilateral pelvic (right)	1	ICG	Cervix	Unilateral	No	2 (left)	–	8	Serous	Detected by algorithm
Algorithm Sensitivity: 93.7 % (75/80); Algorithm FNR: 6.2% (5/80)											

*SLN* sentinel lymph node, *ITC* isolated tumor cells, *LND* lymph node dissection, *FNR* false negative rate, *pN0* pathologic node negative patients, *pN0*(*i+*) pathologic isolated tumor cell in SLN, *pN1*(*mi*) pathologic micrometastasis in SLN, *pN1* pathologic macrometastasis

**Fig. 2:**
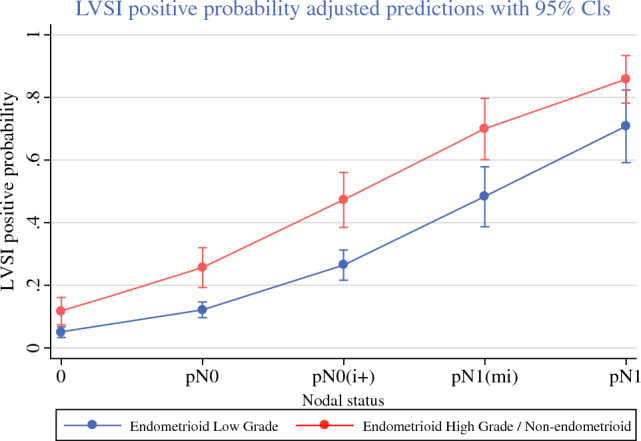
LVSI probability according to the volume of disease in SLN

When assessing factors associated to the detection of disease in the SLN, we identified high CA125, myometrial invasion > 50%, and presence of LVSI as predictors of SLN disease in multivariant analysis (Tables 3, 4). In fact, we observed a close correlation between the volume of nodal disease in SLN and the presence of LVSI in surgical specimen, and this association was evident in low-grade and high-grade tumors (Fig. 2).

**Table 3:** Univariate and multivariate analysis of risk factors associated to disease in SLN

Variable	Univariate analysis		Multivariate analyisis	
	OR CI 95%	*p* value	OR CI 95%	*p* value
Age	1.03 (1.01–1.05)	0.004	1.01 (0.98–1.04)	0.473
CA125	1.02 (1.01–1.03)	< 0.001	1.01 (1.00–1.02)	0.001
Non-endometrioid histology	1.72 (1.00–2.95)	0.052		
Myometrial invasion > 50%	3.48 (2.27–5.34)	< 0.001	2.23 (1.16–4.31)	0.017
High grade (G3)	1.46 (0.91–2.35)	0.115		
LVSI	8.47 (5.41–13.3)	< 0.001	5.39 (2.85–10.18)	< 0.001

**Table 4:** Univariate and multivariate analysis of risk factors associated to cancer-specific survival

Variable	Univariate analysis		Multivariate analyisis	
	HR CI 95%	*p* value	HR CI 95%	*p* value
Nodal status	Reference		Reference	
Negative				
Micrometastasis + ITC	1.37 (0.18–10.54)	0.762	0.31 (0.03–3.10)	0.318
Macrometastasis	5.33 (1.88–15.15)	0.002	0.44 (0.10–1.92)	0.272
Age	1.09 (1.03–1.14)	0.001	1.07 (1.00–1.12)	0.028
CA125	1.01 (1.00–1.01)	0.088		
Non-endometrioid histology	8.00 (3.15–20.3)	< 0.001	1.45 (0.46–4.59)	0.525
FIGO 2009 stage				
I–II	Reference		Reference	
III–IV	8.20 (3.24–20.79)	< 0.001	6.51 (1.85–22.86)	0.003
High grade (G3)	21.2 (6.14–73.3)	< 0.001	10.4 (2.2–49.8)	0.003
LVSI	6.20 (2.40–16.03)	< 0.001	2.77 (0.91–8.46)	0.074
Adjuvant treatment	12.14 (1.62–91.27)	0.015	0.98 (0.10–9.50)	0.989

### Oncologic Outcomes according to Nodal Disease

**Fig. 3:**
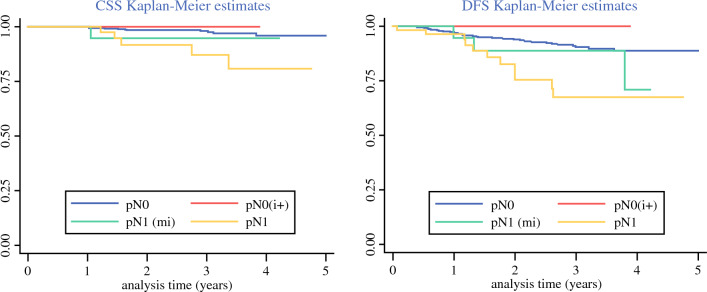
Cancer-specific survival and disease-free survival estimates of node-negative patients (pN0), patients with ITC [pN0(i+)], patients with micrometastases [pN1(mi)], and patients with macrometastases (pN1)

Regarding adjuvant treatment, the use of brachytherapy (BT) was similar across the global cohort and independent of nodal status. We observed a significant higher use of chemotherapy and external beam radiotherapy in patients with micrometastases and macrometastases (Table 5). In this context of adjuvant treatment, and after a median follow up of 1.81 (IQR 0.91–2.97) years, cancer-specific survival and disease-free survival were significantly lower in patients who had macrometastases when compared with patients who had not nodal disease or with low-volume disease only (Fig. 3).

**Table 5:** Adjuvant treatment and survival outcomes according to nodal disease

Variable, *n* (%), median (IQR)	pN0 (*n* = 784)	pN0(i+) (*n* = 25)	pN1(mi) (*n* = 24)	pN1 (*n* = 59)	Total (*n* = 892)	*p* value
*Adjuvant treatment*						
Received adjuvant treatment	391 (49.9)	16 (64.0)	24 (100.0)	58 (98.31)	489 (54.8)	< 0.001
Radiotherapy	376 (48.0)	15 (60.0)	22 (91.7)	51 (86.4)	464 (52.0)	< 0.001
EBRT*	125 (33.2)	5 (33.3)	19 (86.4)	47 (92.2)	196 (42.2)	< 0.001
Brachytherapy*	341 (90.7)	14 (93.3)	17 (77.3)	42 (82.4)	414 (89.2)	0.068
Chemotherapy	90 (11.5)	2 (8.0)	13 (54.2)	48 (81.4)	153 (17.2)	< 0.001
Concurrent chemoradiotherapy	1 (0.1)	1 (4.0)	2 (8.3)	7 (11.9)	11 (1.2)	< 0.001
*Survival*						
Median follow-up (years)	1.82 (0.92–0.97)	1.25 (0.70–2.37)	2.29 (2.34–1.05)	1.60 (0.80–3.08)	1.81 (0.91–2.97)	0.092
OS 3y	95.8 (93.2–97.4)	100 (NC–NC)	94.1 (65.0–99.2)	84.8 (66.6–93.5)	95.1 (92.6–96.7)	0.034
DFS 3y	90.8 (87.4–93.3)	100 (NC–NC)	89.1 (62.5–97.2)	68.0 (48.5–81.3)	89.3 (86.0–91.9)	0.001
CSS 3y	98.0 (96.2–99.0)	100 (NC–NC)	94.1 (65.0–99.2)	87.3 (69.0–95.2)	97.2 (95.2–98.4)	0.005
*Status at last control*						
Alive without disease	739 (94.3)	25 (100.0)	21 (87.5)	51 (86.4)	836 (93.7)	0.052
Alive with disease	23 (2.9)	0 (0.0)	2 (8.3)	2 (3.4)	27 (3.0)	
Dead	22 (2.8)	0 (0.0)	1 (4.2)	6 (10.2)	29 (3.3)	
*Death caused by disease*	11 (50.0)	0 (0.0)	1 (100.0)	5 (83.3)	17 (58.6)	
*Other causes for death*	11 (50.0)	0 (0.0)	0 (0.0)	1 (16.7)	12 (41.4)	

^*^Percentages are calculated over the patients who received radiotherapy as adjuvant treatment

*EBRT* external beam radiotherapy, *OS* overall survival, *DFS* disease free survival, *CSS* cancer-specific survival, *pN0* pathologic node negative patients, *pN0*(*i+*) pathologic isolated tumor cell in SLN, *pN1*(*mi*) pathologic micrometastasis in SLN, *pN1* pathologic macrometastasis

When analyzing risk factors associated to cancer-specific survival in our cohort, only older age, high-grade histology, and FIGO 2009 stages III–IV remained as significant in multivariate analysis (Table 4).

## Discussion

We describe the results of a retrospective, multi-institutional cohort of patients with early stage EC studied with SLN biopsy as a part of their surgical treatment. Patients included in this study received their surgical treatment when SLN biopsy was not a wide recommendation in clinical guidelines^25^ and the best detection technique had not yet been established.^2^ Therefore, the injection site and tracers used for SLN mapping were in some cases different from the most recent recommendations. In these patients, SLN technique was performed by different surgical teams, according to each center’s protocol and facilities, making these results representative of the implementation of SLN biopsy in a real clinical scenario. In addition, this cohort includes the learning curve of most of the Spanish centers in this technique. However, we reach high rates of sensitivity and low false negative rates. Interestingly, 22% of patients with macrometastasis in SLN and 50% of patients with micrometastasis had been classified as patients at low risk of nodal disease according to their preoperative characteristics (histology, myometrial infiltration, cervical invasion). In former clinical guidelines,^25^ these patients were not candidates to receive any nodal assessment and would not have been adequately diagnosed, therefore revealing the importance of SLN biopsy implementation in the management of patients with early stage EC.

We report a rate of positive SLN of 9.3% after excluding ITCs, probably related to the fact that less than 15% of the cohort was considered at high risk of nodal disease preoperatively, and most of the patients were at low and intermediate risk. These results are in agreement with previously published data. The review and meta-analysis recently published by the Cochrane Collaboration^26^ including 2237 women, reported rates of positive lymph nodes (excluding ITCs) ranging from 5.2% to 34.4% with a mean of 20.1% (95% CI 17.7–22.3%).

The high number of patients with lymphadenectomies performed (47% pelvic and 25% aortic) allows for the calculation of the accuracy of SLN technique when used in a clinical setting. After applying the algorithm, we obtained a sensitivity of 93.7% and a false negative rate of 6.2%, which is in line with the results of other studies.^19^ We found a rate of isolated aortic metastases of 1.6%, similar to previously reported results.^9^ The high proportion of low-volume disease in this cohort (45%) is also in agreement with previous reported cohorts (54% in FIRES trial,^7^ 67% in FILM trial^8^).

Even though the rate of low-volume disease may change depending on the technique used for SLN ultrastaging,^27^ it is indisputable that any ultrastaging method will lead to the detection of patients bearing low-volume disease in SLN in a high number. Defining the best adjuvant management for these patients is a clinical need when implementing SLN biopsy as an effective tool of lymph node evaluation in EC. In the present study, the use of pelvic external beam radiotherapy, chemotherapy, or both was almost universal among patients who had micrometastases or macrometastases. Patients with ITC in SLN received adjuvant treatment similarly to patients who had negative nodes, as recommended by most recent clinical guidelines.^2^ In this context, patients with low-volume disease (ITC and micrometastases) had similar results in terms of overall and disease-free survival than patients with negative nodes. These findings had been previously reported by other authors and are the basis for the recommendation of not to treat as node positive the patients that only present ITC in SLN biopsy.^15,16^

In our cohort, patients with micrometastases showed an excellent prognosis after being treated as node-positive patients, and their outcome was more favorable than patients with macrometastases. Ignatov et al. reported the outcomes of 95 patients with micrometastases who received adjuvant treatment and 31 patients with micrometastases without adjuvant treatment. Without adjuvant therapy, disease-free survival was significantly reduced as compared with disease-free survival in the node-negative cohort (*p* = 0.0001). With adjuvant therapy, the median disease-free survival of patients was similar to node-negative patients (*p* = 0.648).^22^ Despite growing evidence as to the prognostic significance of micrometastases, the clinical relevance of ITC on SLN is still a matter of debate. Ghoniem et al. reported one of the largest series of patients with ITC detected on SLN, including 132 patients. Of them, 47 patients with endometrioid histology did not receive any adjuvant treatment or only vaginal brachiterapy. Patients with endometrioid grade 1 disease and neither LVSI nor uterine serosal involvement showed low recurrence rates despite the omission of adjuvant treatment. However, the authors were cautious with these results, as longer follow-up of these patients was needed.^16^

We identified older age, advanced FIGO stage, and high-grade histology as factors associated to decreased cancer-specific survival. Non-endometrioid histology, LVSI, and uterine serosal invasion had also been previously reported as independent predictors of recurrence in patients with low-volume disease on SLN.^16^ We also observed that LVSI behaves as a potent predictor of SLN disease. This finding highlights the importance of LVSI as a prognostic marker in EC and reveals that more efforts should be made to identify preoperative surrogates of LVSI that could detect patients at higher risk of nodal disease.

### Strengths and Limitations

This is the first study evaluating the outcomes of patients undergoing SLN mapping for early stage endometrial cancer across many different centers in Spain. The number of cases included provides good statistical power, but the retrospective nature of the study entails biases that should be considered. The median follow-up period is close but does not exceed 2 years, which is considered the time of higher risk of relapse and could be a limitation of the study. The surgical approach, technique used for SLN detection, and ultrastaging protocols were dependent on each center, although we consider this as a strength of the study given that it reflects the real clinical implementation of SLN biopsy according to technological availability and team competencies. Nevertheless, some steps to increase detection rates and reduce the false negative rate must be taken, such as increasing the experience of surgical teams, maximizing the use of ICG technology, and improving ultrastaging techniques.

## Conclusions

In our nationwide cohort on SLN biopsy for initial-stage endometrial cancer, we achieved a sensitivity of 93.7% and a false negative rate of 6.2% for detecting nodal disease. We identified 12.1% of patients with positive lymph nodes, 45.3% of them with low-volume disease (micrometastases and ITCs) only. Patients with micrometastases showed excellent outcomes after receiving adjuvant treatment as node-positive patients. Patients with ITC showed excellent outcomes after receiving adjuvant treatment as node-negative patients. Age, high-grade histology, and FIGO 2009 stages III–IV were factors that negatively impacted cancer-specific survival. The presence of LVSI in surgical specimen was strongly correlated with the volume of the disease found on SLN.
